# Heparan Sulfate Proteoglycans: Key Mediators of Stem Cell Function

**DOI:** 10.3389/fcell.2020.581213

**Published:** 2020-11-19

**Authors:** Maanasa Ravikumar, Raymond Alexander Alfred Smith, Victor Nurcombe, Simon M. Cool

**Affiliations:** ^1^Glycotherapeutics Group, Institute of Medical Biology, Agency for Science, Technology and Research (A^∗^STAR), Singapore, Singapore; ^2^Department of Orthopaedic Surgery, Yong Loo Lin School of Medicine, National University of Singapore, Singapore, Singapore; ^3^Lee Kong Chian School of Medicine, Nanyang Technological University–Imperial College London, Singapore, Singapore

**Keywords:** proteoglycans, GAGs, stem cells, embryonic stem cells, adult stem cells, extracellular matrix, glycosaminoglycan, heparan sulfate

## Abstract

Heparan sulfate proteoglycans (HSPGs) are an evolutionarily ancient subclass of glycoproteins with exquisite structural complexity. They are ubiquitously expressed across tissues and have been found to exert a multitude of effects on cell behavior and the surrounding microenvironment. Evidence has shown that heterogeneity in HSPG composition is crucial to its functions as an essential scaffolding component in the extracellular matrix as well as a vital cell surface signaling co-receptor. Here, we provide an overview of the significance of HSPGs as essential regulators of stem cell function. We discuss the various roles of HSPGs in distinct stem cell types during key physiological events, from development through to tissue homeostasis and regeneration. The contribution of aberrant HSPG production to altered stem cell properties and dysregulated cellular homeostasis characteristic of cancer is also reviewed. Finally, we consider approaches to better understand and exploit the multifaceted functions of HSPGs in influencing stem cell characteristics for cell therapy and associated culture expansion strategies.

## Introduction

The glycocalyx is a dense layer of glycoproteins and glycolipids that coats the exterior of the cell membrane. It is comprised of a variety of polysaccharides covalently attached to lipids or proteins, and functions as an essential interface between external and internal cellular environments. Heparan sulfate (HS) is one of the many constituent polysaccharides, and is long (40–300 residues), linear and highly charged (Sarrazin et al., 2011). HS belongs to the glycosaminoglycan (GAG) family of carbohydrates, which also includes the closely related heparin, hyaluronan (HA), chondroitin and dermatan sulfate (CS/DS), and keratan sulfate (KS) (Esko and Lindahl, 2001; Gallagher, 2001). The base structure of HS is highly conserved throughout multicellular organisms, including mammals, fruit flies (*Drosophila melanogaster*) (Nakato et al., 1995), nematodes (*Caenorhabditis elegans*) (Townley and Bülow, 2011) and hydra (*Hydra Magnipapillata* and *Hydra vulgaris*) (Sarras et al., 1991; Yamada et al., 2007), indicating an ancient evolutionary origin.

HS is present in the extracellular matrix (ECM) of every tissue and on the surface of virtually every cell within an organism. HS chains are covalently attached to core proteins belonging to multiple families, and are collectively known as heparan sulfate proteoglycans (HSPGs). Mammalian HSPGs may be present as transmembrane proteins (syndecan 1–4, CD44, neuropilin-1, betaglycan) (Rapraeger et al., 1985; Sanderson and Bernfield, 1988; Andres et al., 1989; Brown et al., 1991; Kojima et al., 1992; Shintani et al., 2006; Multhaupt et al., 2009; Couchman, 2010; Gopal et al., 2010), GPI-anchored membrane proteins (glypican 1–6) (David et al., 1990; Filmus and Selleck, 2001; Filmus et al., 2008; Feil et al., 2009), secreted proteins retained within the ECM (perlecan, agrin, and collagen XVIII) (Noonan et al., 1991; Tsen et al., 1995; Halfter et al., 1998; Knox et al., 2002; Knox and Whitelock, 2006) or in secretory granules (serglycin) (Tantravahi et al., 1986; Stevens et al., 1988; Kolset and Tveit, 2008).

The ubiquitous presence of HSPGs is indicative of the multitude of important biological roles they play within an organism. For example, HS-rich perlecan is an integral basement membrane constituent, along with agrin and collagen XVIII, and a critical scaffolding component of the ECM (Hassell et al., 1980; Costell et al., 1999; Farach-Carson and Carson, 2007). In this regard, HSPGs provide biomechanical support and help maintain integrity of the extracellular microenvironment. Apart from their structural functions, HS chains can bind to a large number of proteins, of which at least 437 have been identified, and serve as low-affinity co-receptors during signaling events (Xu and Esko, 2014; Rudd et al., 2017; Dubey et al., 2020). Additionally, the protein-binding properties of HS afford it the ability to arrange morphogens into spatial and temporal gradients, which is vital for orderly development and tissue repair (Bernfield et al., 1999). HS can also bind and sequester various proteins, protecting them from thermal or proteolytic degradation (Sadir et al., 2004; Makarenkova et al., 2009; Xu and Esko, 2014; Kjellén and Lindahl, 2018; Poon et al., 2018; Sun C. et al., 2019). The distinct combinations of functional groups and chain modifications laid down during biosynthesis confer unique structural conformations and binding properties to an HS chain, underpinning its propensity to interact with a wide array of ligands and cognate receptors (Kreuger et al., 2006; Lindahl and Li, 2009).

HSPGs may also interact with proteins via the cytoplasmic domains of their core proteins. The syndecans are especially known to engage components of the cytoskeleton and focal adhesions, such as α-actinin and integrins, despite an absence of inherent kinase activity (Rapraeger et al., 1986; Saoncella et al., 1999; Greene et al., 2003). Such interactions implicate HSPGs as crucial components of protein binding events not only at the cell surface, but also those within adjacent intracellular regions that may influence cytoskeletal organization and downstream signaling cascades. All of these functional features confer an indispensability of HSPGs in cellular responses that influence cell morphology, adhesion, migration, and fate decisions (Longley et al., 1999; Cool and Nurcombe, 2006; Gopal et al., 2010; Bass et al., 2011; Okina et al., 2012; Vuong et al., 2015; Mitsou et al., 2017). In this review, we highlight some of the intricate structural features of HS and their proteoglycans (PGs) that underpin such functionality, and discuss the importance of specific HSPG signatures in mediating the behavior of different stem cell types.

## HS Structure and Biosynthesis

**FIGURE 1:**
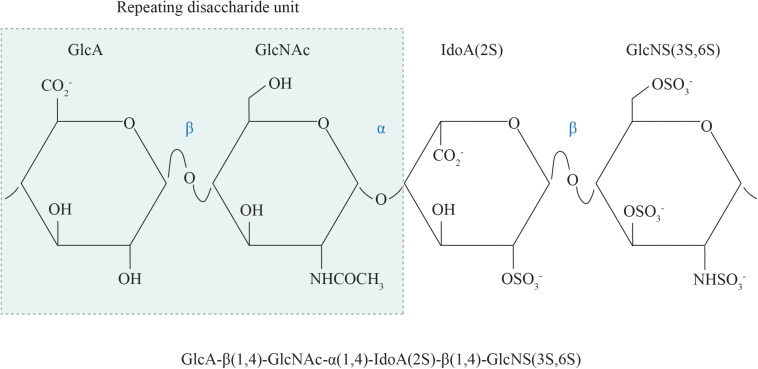
The chemical structure of HS. The repeating disaccharide unit of HS comprises a GlcA residue linked to GlcNAc residue via a β1,4 glycosidic bond. Selective GlcA monosaccharides undergo epimerization to IdoA, which is particularly susceptible to sulfation at the C2 position. Sulfate moieties may also be added to the *N*, C6 and rarely, C3 positions of the glucosamine residue. GlcA, glucuronic acid; GlcNAc, *N*-acetyl glucosamine; IdoA, iduronic acid; GlcNS, *N*-sulfated glucosamine; 2S, 2-*O*-sulfation; 6S, 6-*O*-sulfation; 3S, 3-*O*-sulfation.

**FIGURE 2:**
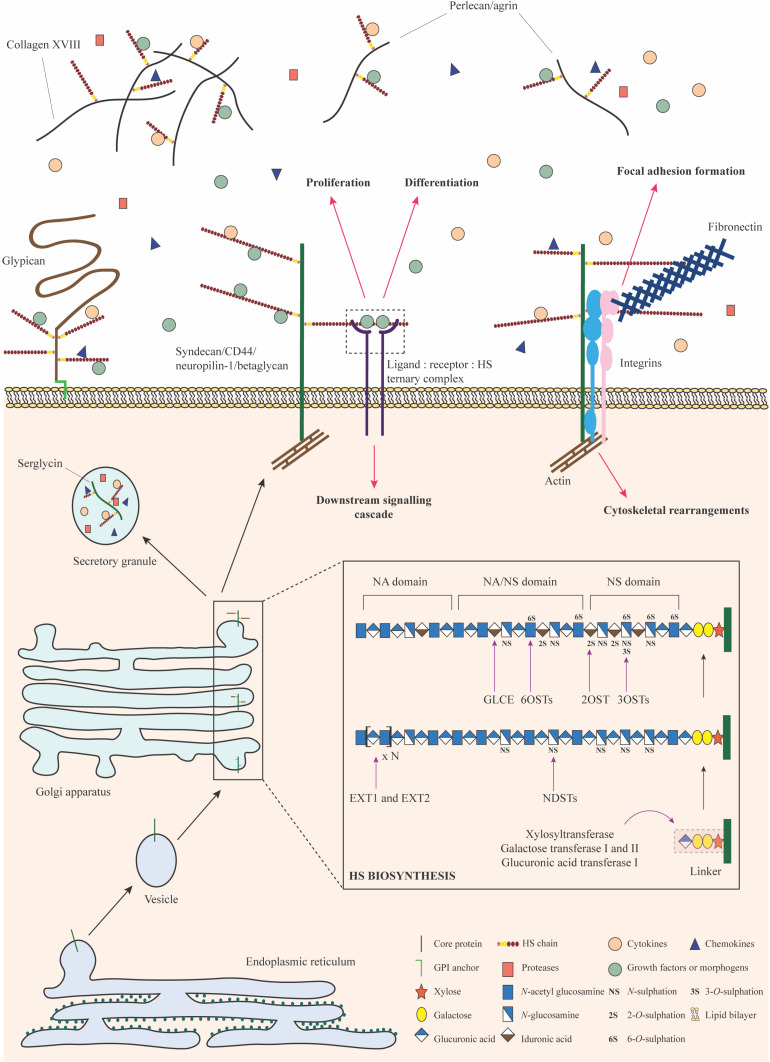
HSPG biosynthesis and functions. HS biosynthesis is a sequential process that occurs in the Golgi apparatus. It is initiated by the formation and attachment of a tetrasaccharide linker to a serine residue on the core protein. Subsequently, chain polymerization is carried out by EXT1 and EXT2, where *N*-acetylglucosamine and glucuronic acid residues are added in succession. The HS chain is then modified by a collection of enzymes including the NDSTs, GLCE, 2OST, 6OSTs, and 3OSTs. Selective sulfation and epimerization confer intricate structural nuances to the HS chain, underpinning the formation of domains with distinct functional features. Upon exit from the Golgi apparatus, HSPGs may be stored in secretory granules, transported to the plasma membrane or secreted into the ECM. They bind to a variety of signaling factors and function as co-receptors to mediate signaling cascades important for stem cell proliferation and differentiation. HSPGs may engage with integrins, fibronectin, and actin filaments to bring about cytoskeletal reorganization for focal adhesion formation or migration. HSPGs can also sequester signaling molecules in the ECM and regulate their bioavailability. Monosaccharides in this figure have been represented in accordance with the symbol nomenclature for glycans (SNFG) (Varki et al., 2015).

The basic structural unit of the HS polysaccharide is that of a repeating disaccharide composed of uronic acid (either glucuronic acid or its C5-epimer, iduronic acid) β1,4 linked to *N*-glucosamine, which may be *N*-acetylated, *N*-sulfated or in rare circumstances, *N*-unsubstituted (free amine) (Figure 1; Esko and Lindahl, 2001). A wide range of structural diversity and functional complexity is conferred upon modification of the constituent monosaccharides—the uronic acid may be sulfated at the C2 position and the glucosamine at the *N*, C6 and rarely, C3 positions. The synthesis of HS is carried out within the Golgi apparatus by a series of biosynthetic enzymes (Figure 2), through the catalysis of nucleotide sugars (such as UDP-glucosamine) and the nucleotide sulfate donor 3′-phosphoadenosine-5′-phosphosulfate (PAPS) (Esko and Lindahl, 2001). The majority of HS biosynthetic enzymes are type II integral Golgi membrane proteins except for 3-*O*-sulphotransferase 1 (3OST1), which possesses an intraluminal resident form (Shworak et al., 1997).

HS chains are attached to PG core proteins as post-translational modifications via a linkage tetrasaccharide. This “linker” sequence is composed of a glucuronic acid residue, two galactose residues and a xylose residue covalently bound to a serine hydroxyl group through a xylosidic bond (Lindahl and Rodén, 1965; Lindahl, 1966). A heterodimeric complex of the HS co-polymerases exostosin 1 and 2 (EXT1 and EXT2) alternatively adds *N*-glucosamine and glucuronic acid residues, resulting in the basic structure and length of the chain. Following this, *N*-deacetylase/*N*-sulphotransferase (NDST; 4 isoforms) removes acetyl groups and adds a sulfate moiety to the *N* position of select glucosamine residues. This process yields short *N*-sulfated domains that serve as substrates for further modification. On rare occasion, deacetylated glucosamine residues may be left as free amine groups (Sheng et al., 2011; Dou et al., 2015). Next, glucuronyl C5-epimerase (GLCE) selectively converts glucuronic acid residues into iduronic acid, a process that requires an adjacent *N*-sulphoglucosamine at the non-reducing end (Hagner-McWhirter et al., 2004). This is followed by 2-*O*-sulfation at the C2 position of the uronic acid, brought about by 2-*O*-sulphotransferase (2OST); this modification occurs predominantly in iduronic acid residues rather than glucuronic acid (Smeds et al., 2010). Subsequently, the C6 positions of select glucosamine residues are sulfated by 6-*O*-sulphotransferase (6OST; 3 isoforms) (Xu et al., 2017). In rare cases, the C3 position may also be sulfated by 3-*O*-sulphotransferase (3OST; 7 isoforms) (Shworak et al., 1999). At this stage, HS is transported to the cell surface where a final modification involving the selective removal of 6-*O*-sulfate groups from glucosamine residues may occur, through the action of extracellular 6-*O*-endosulphatase (SULF; 2 isoforms collectively referred to as “sulfatases”) (Ai et al., 2003).

## The Intricate Structure of HS Imparts Essential Functions

A complete HS chain possesses complex structural heterogeneity seldom observed in most biomolecules. Short, highly sulfated, and heterogeneous regions (NS domains) are flanked by short transition domains of variable sulfation (NA/NS domains) (Figure 2). The NA/NS domains are interconnected by long, flexible regions of little to no modification (NA domains) (Turnbull and Gallagher, 1990, 1991; Maccarana et al., 1996; Gallagher, 2001). HS functionality is imparted predominantly by the highly negatively charged NS domains. The disaccharide residues in these regions are capable of interacting with small hydrophilic clusters of positively charged amino acid residues, such as lysine and arginine, common characteristics observed within the three-dimensional structure of HS-binding proteins (Cardin and Weintraub, 1989; Faham et al., 1996; Rudd et al., 2017). Interactions of HS with the fibroblast growth factor (FGF) family and their cognate receptors (FGFRs) serve as quintessential examples of this feature, which underpins the role of HSPGs as low-affinity co-receptors (Rapraeger et al., 1991; Yayon et al., 1991; Nurcombe et al., 1993).

A high affinity binding site for FGF2 in fibroblast-derived HS was discovered to be an oligosaccharide that is 7 disaccharides in length or has a degree of polymerization of 7 (dp7) (Turnbull et al., 1992). This dp7 fragment and other longer heparin/HS-derived oligosaccharides containing the dp7 sequence were characterized by an enrichment of 2-*O*-sulfated iduronic acid (IdoA2S) and *N*-sulfated glucosamine (GlcNS) residues (Turnbull et al., 1992; Ishihara et al., 1994; Kreuger et al., 1999). The necessity of these IdoA2S and GlcNS residues for FGF2 binding was evidenced when treatment of the dp7 fragment with heparinase led to an abolishment of FGF2 binding. Moreover, other similarly sized oligosaccharides with reduced 2-*O*-sulfation exhibited lower affinities for FGF2, highlighting the requirement of contiguous stretches of highly sulfated disaccharides for strong FGF2 binding (Turnbull et al., 1992). Further enquiry into the compositional features of HS and heparin oligosaccharides that are required for FGF interactions has shown that the FGFs bind to certain shared HS epitopes with low affinity and selectivity (Kreuger et al., 2005). However, distinct sulfation patterns and chain lengths allow for the formation of specialized NS domains in the GAG chain, which preferentially bind specific FGFs with a higher affinity than others (Guimond and Turnbull, 1999; Schultz et al., 2017). For example, while IdoA2S and GlcNS residues are essential for FGF2 binding, additional 6-*O*-sulfation of GlcNS residues (GlcNS6S) is required for high affinity binding to FGF1 and FGF4 (Ishihara, 1994; Kreuger et al., 1999; Ashikari-Hada et al., 2004). The binding affinity of HS/heparin oligosaccharides to FGF1 and FGF4 is also reported to correlate with chain length. Kreuger et al. (1999) have shown that the minimum length required for a heparin oligosaccharide to exhibit binding affinity to FGF1 is dp6. However, dp10 fragments and larger are needed for tight binding with both FGF1 and FGF4 (Ishihara, 1994; Kreuger et al., 1999).

In addition to FGF binding, HS chains bind to corresponding FGFRs to enhance ligand–receptor interactions and promote receptor dimerization for ternary complex formation (Ornitz et al., 1992; Spivak-Kroizman et al., 1994; Pellegrini et al., 2000; Schlessinger et al., 2000). While 6-*O*-sulfate moieties on glucosamine residues of HS/heparin are dispensable for FGF2 binding (Guimond et al., 1993; Maccarana et al., 1993; Ishihara et al., 1994), they are required for engaging FGFR, facilitating ternary complex formation and subsequently, activating the mitogenic effect of FGF2–FGFR interactions (Ostrovsky et al., 2002). In a study by Guimond and colleagues, exogenous full-length heparin was capable of rescuing the mitogenic effect of FGF2 in fibroblasts devoid of HS. However, selective 6-*O*-desulfation of heparin inactivated FGF2-induced mitogenesis, even though the oligosaccharide was able to bind FGF2 and compete with endogenous heparin for FGF2 binding (Guimond et al., 1993). The requirement of 6-*O*-sulfate moieties for activating FGF2-dependent mitogenesis upon HS/heparin binding was further established when disaccharide analysis of a range of different HS oligosaccharides clearly showed that the major distinguishing factor between activating and inhibitory fragments was the extent of 6-*O*-sulfation (Pye et al., 1998). Apart from appropriately 6-*O*-sulfated residues, chain length was also found to be an important factor for determining the ability of a HS/heparin oligosaccharide to support FGF2 mitogenic activity. An exogenous heparin-derived dodecasaccharide (dp12) was comparable to full-length heparin in rescuing the mitogenic effect of FGF2 in fibroblasts devoid of HS, whereas HS/heparin fragments lower than dp10 lacked the ability to support this activity (Guimond et al., 1993; Ishihara, 1994; Pye et al., 1998). Therefore, the structural and compositional features of HS heavily influence the functional properties of the chain, and consequently, the ability of HSPGs to operate effectively as signaling co-receptors (Goodger et al., 2008; Jastrebova et al., 2010).

## Heterogeneity in HSPG Composition and Localization Can Influence Stem Cell Fate

Structural and functional heterogeneity in HSPGs has been observed between different cell types and within particular cell populations over time (Turnbull and Gallagher, 1988; Allen et al., 2001). The HSPGs produced by a cell (heparanome) can be rapidly altered with changes in cell state. Such alterations can influence cell activity in distinct ways depending on the compositional complexity of the heparanome (Turnbull et al., 2001).

HSPG heterogeneity can be seen at both the GAG chain and core protein levels. Heterogeneity at the HS chain level has largely been attributed to the non-template and incomplete nature of the HS biosynthetic process, as well as the activity of multiple biosynthetic enzyme isoforms that may differ in their substrate specificities and the products they generate (Aikawa and Esko, 1999; Liu et al., 1999; Habuchi et al., 2000; Turnbull et al., 2001). Post-biosynthetic alterations to sulfation and chain length are also known to contribute to the structural diversity and associated functional variability in HS. For example, selective removal of 6-*O*-sulfate groups by the SULF enzymes, SULF2 in particular, may lead to domain reorganization within the HS chain, prevent the binding of HS to signaling factors and consequently, alter ligand–receptor interactions that influence stem cell or progenitor cell fate decisions (Ai et al., 2003; Lai et al., 2003; Hammond et al., 2014). The drastic effects that a lack of appropriate HS moieties can have on signaling and progenitor cell function was examined in MM14 myoblast cultures *in vitro*—desulfation of cell surface HS by heparinase or chlorate treatment prevented FGF2 binding to cognate cell surface receptors and removed the suppressive effect of FGF2 on myoblast differentiation (Rapraeger et al., 1991). A similar trend was observed with the addition of selectively 6-*O*-desulfated heparin to MM14 cultures. The heparin oligosaccharides competed with endogenous HS for FGF2 binding and precluded FGF2–FGFR interactions that normally suppress cell cycle exit and myogenic activation (Guimond et al., 1993). The cleavage of 6-*O*-sulfates from cell surface and secreted HSPGs can also lead to the release of previously bound signaling factors and an increase in the bioavailability of these factors within the ECM (Uchimura et al., 2006; Rosen and Lemjabbar-Alaoui, 2010). Alternatively, the biodistribution of signaling molecules in the ECM may be altered as a result of HS chain cleavage by the single copy heparanase enzyme (HPSE), since cleavage releases biologically active HS fragments that are capable of binding and sequestering signaling factors within the extracellular space (Kussie et al., 1999; Hulett et al., 2000; Vlodavsky et al., 2002).

In the case of core proteins, heterogeneity can result from the differential expression of encoding genes. Spatiotemporal variability in the expression of core proteins, especially during development and wound healing, can influence signaling events at the cell surface and affect a stem cell’s responsiveness to its surroundings (Bernfield et al., 1993; Olczyk et al., 2015). The collection of core proteins present at the cell surface and their localization may also be altered upon cleavage of PG ectodomains in a process known as “shedding” (Nam and Park, 2012). Studies have shown that shedding is mediated by a variety of proteases and phospholipases, collectively known as “sheddases,” which can exhibit specificity toward particular core proteins. For example, the syndecans are known targets of a number of matrix metalloproteinases (MMPs), such as MMP7, MMP9, and ADAM17 (Ding et al., 2005; Brule et al., 2006; Pruessmeyer et al., 2010). Recently, ADAM17 has also been reported as a glypican sheddase, in addition to conventional phospholipases, such as Notum, that cleave at the GPI anchor (Traister et al., 2008; Kawahara et al., 2017). The shedding process yields bioactive soluble forms of HSPGs in the ECM that act as paracrine or autocrine effectors, and compete against cell-surface/matrix-bound HS (Nam and Park, 2012). The intact HS chains attached to the cleaved PG ectodomains can also operate as reservoirs that bind signaling molecules and regulate their diffusion, aiding in the maintenance of morphogen gradients that direct stem cell responses (Yan and Lin, 2009).

Heterogeneity in chain composition, core protein expression and PG localization underpin a highly diverse heparanome, which may directly influence the repertoire of molecular cues within a cell’s microenvironment as well as the cell’s ability to respond to them. However, there are several important questions yet to be answered regarding this diversity, especially pertaining to potential differences in functionality between cell surface HSPGs, cleaved or shed HSPGs, and secreted HSPGs. It remains to be understood whether HSPGs vary in the HS chains they carry depending on the core protein type and positional status. Current experimental methods to examine HSPG composition and function involve the extraction of PG pools from cell cultures or tissues and therefore, lack the resolution to interrogate distinct HSPG fractions or individual HSPGs. Such an inability to probe potentially unique compositional characteristics and mechanisms of action of specific HSPGs presents a major challenge in the field, and has hindered an understanding of fine functional nuances that may exist between HSPGs.

## HSPGs as Orchestrators of Development and Differentiation

### Lessons From Knockout Mouse Models

Over the past two decades, genetic modification has been one of the primary methods used for investigating the functions of HS modifications in mediating stem cell fate during development and differentiation. The knockout (KO) of individual genes from the HS biosynthetic pathway has revealed essential roles played by distinct modifications in tuning cellular responses to GFs, cytokines, chemokines, and morphogens.

#### *Ext1* Knockout

In 2000, Lin and colleagues first developed the *Ext1*^–/–^ mouse, which was devoid of HS due to the loss of the HS co-polymerase EXT1 (Lin et al., 2000). *Ext1*^–/–^ embryos failed to develop beyond the blastocyst stage, clearly indicating that HS is an essential regulator of gastrulation and embryogenesis. Analysis of *Ext1*^–/–^ embryonic stem cell (ESC) behavior *in vitro* has been carried out extensively by the Merry lab, specifically with regards to the development of the neurectoderm and mesoderm (Johnson et al., 2007; Baldwin et al., 2008; Holley et al., 2011; Pickford et al., 2011; Meade et al., 2013). Over the course of several studies, the essential regulatory role of HS in the exit of murine ESCs (mESCs) from pluripotency was discovered; *Ext1*^–/–^ ESCs were incapable of exiting the pluripotent state under neural (Johnson et al., 2007; Pickford et al., 2011; Meade et al., 2013) or mesodermal (Holley et al., 2011) differentiation conditions, instead retaining characteristics of pluripotent cells, such as high levels of OCT4 expression. Interestingly, the addition of exogenous heparin or HS was able to rescue differentiation in these studies, further indicating that HS is a crucial regulator of stem cell function.

These findings were further validated in two studies by Kraushaar et al. (2010, 2012), who revealed that *Ext1*^–/–^ mESCs were capable of maintaining pluripotency despite the removal of leukemia inhibitory factor. The absence of HS resulted in defective FGF and BMP signaling, such that the KO mESCs were unable to respond to pro-differentiation factors. Additional studies have revealed potential compensatory mechanisms by which the levels of other sulfated GAGs (such as CS and DS) appear to be upregulated in *Ext1*- null cells. This idea was initially proposed in a study by Le Jan and colleagues, wherein *Ext1*^–/–^ embryoid bodies (EBs; spheroid cell aggregates capable of crudely recapitulating early embryonic development) displayed increased levels of CS/DS, and were able to respond to VEGF as well as undergo sprouting angiogenesis despite the loss of HS (Le Jan et al., 2012). The existence of putative compensatory mechanisms between different GAG species and consequent effects on signaling cascades suggests yet another layer of complexity in the GAG-dependent modulation of stem cell behavior, which is yet to be fully explored.

#### *Ndst1/2* Knockout

Whilst the loss of *Ext1*^–/–^ highlights the absolute requirement of HS in development and differentiation, it does little to reveal how changes in specific modifications within the HS chain, such as *N*-sulfation, within the HS chain could affect the behavior of cells. As described previously, NDST1 and 2 are primarily responsible for laying down *N*-sulfate modifications and forming NS domains within HS (NDST1) and heparin (NDST2) (Forsberg and Kjellén, 2001). In 2004, Holmborn and colleagues derived *Ndst1/2*^–/–^ mESCs; analysis of the HS produced in these cells indicated a total loss of *N*- and 2-*O*-sulfation. Interestingly, 6-*O*-sulfation was still present and transcripts for all three 6OSTs were expressed, with 6OST1 displaying the greatest transcript abundance (Holmborn et al., 2004). Although functional assays were not performed, it was revealed that the presence of NS domains was not a pre-requisite for 6-*O*-sulfation. This important observation may be attributed to the unique orientation in which the 6OSTs engage with the HS chain, which is markedly different to that adopted by other sulphotransferases (Xu et al., 2017).

A number of other studies have sought to better elucidate the role of HS *N*-sulfation in early differentiation events and maintenance of pluripotency through use of the *Ndst1/2*^–/–^ mESC line. The loss of *N*-sulfation results in perturbed FGF4 signaling, which in turn manifests as a similar phenotype to *Ext1*^–/–^ mESCs: an inability to exit the pluripotent state (Lanner et al., 2010). Subsequent investigation indicated that despite the loss of *N*-sulfation, *Ndst1/2*^–/–^ mESCs were still able to respond to stimulation by BMP4 (Forsberg et al., 2012). This finding clearly reflected the differential nature and context-dependent role of HS in FGF and BMP signaling. HS is not essential for BMP:BMPRI complex formation, as is the case for FGF:FGFR. Instead, HS binds to BMPRII and enhances its recruitment to BMP:BMPRI complexes, facilitating receptor hetero-oligomerisation for signaling (Kuo et al., 2010). Recent studies have suggested that HS is also able to antagonize BMP signaling (Mundy et al., 2018), offering an insight into why *Ndst1/2*^–/–^ mESCs are still capable of responding to BMP4 and differentiating down an osteoblastic lineage.

Further studies have revealed an essential requirement for *N*-sulfation in angiogenic sprouting and the response to VEGF (Jakobsson et al., 2006). Lack of *N*-sulfation prevented *Ndst1/2*^–/–^ EBs from undergoing angiogenesis. However, chimeric EBs formed with *Vegfr2*^–/–^ mESCs were able to effectively respond to VEGF. This indicated that HS in *trans* was capable of potentiating VEGF signaling and was sufficient to rescue the differentiation capacity of cells devoid of *N*-sulfated residues.

### Embryonic Stem Cells

Preliminary studies on HS expression and function in ESCs focused on mESCs. Immortal, easy to culture and rapidly proliferating, these cells were ideal for obtaining sufficient quantities of biological material to conduct detailed analyses of HS and its biosynthetic pathway. Nairn et al. (2007) used mESCs to study changes in GAG content and composition during differentiation to EBs and extra embryonic endoderm. Findings indicated that HS synthesis was upregulated, together with that of HA and CS/DS, during differentiation. Overall GAG sulfation increased as cells differentiated, with particularly high levels of *N*-sulfated HS disaccharides and di-sulfated CS type E disaccharides (Nairn et al., 2007).

Further studies from the Merry lab focused on early lineage commitment through the use of well-defined differentiation protocols aimed at recapitulating early germ layer specification (Smith et al., 2011). In 2007, an mESC Sox1-green fluorescence protein (GFP) reporter line, 46C, was used to analyze changes in HS sulfation through the course of differentiation to neural progenitor cells (NPCs) (Johnson et al., 2007). The study reported a clear rise in HS sulfation as cells exited a pluripotent state and differentiated into NPCs. Increases in HS biosynthetic enzyme transcripts, FGF2 cell surface binding and *N*, 6- and 2-*O*-sulfated disaccharides were also observed. A similar study in 2008 assessed the changes in HS expression through the course of early mesoderm commitment in mESCs by using an epitope-specific single chain fragment-variable antibody (HS4C3) (Baldwin et al., 2008). During mesodermal differentiation toward the haemangioblast lineage, cells upregulated the HS4C3 epitope [previously identified as containing 3-*O*-sulfation (ten Dam et al., 2006)], coinciding with an upregulation in the transcription factor brachyury. Isolation and reaggregation of cells with high expression of the HS4C3 epitope and brachyury yielded a higher efficiency of differentiation into haemangioblasts, indicating that specific changes in HS fine structure are correlated with enhanced commitment to a particular mature lineage (Baldwin et al., 2008). This was further confirmed via immunocytochemical staining of sectioned primitive streak stage mouse embryos, which displayed high expression of the HS4C3 epitope in the developing mesoderm during gastrulation (Baldwin et al., 2008).

Hirano et al. (2012) proposed that the HS4C3 epitope influenced Fas signaling during mESC differentiation, with overexpression of 3-*O*-sulfation leading to enhanced Fas signaling and differentiation. Conversely, removal of 3-*O*-sulfation reduced Fas signaling and the differentiation potential of mESCs. A follow-up study showed that Fas signaling via the 3-*O*-sulfated epitope recognized by HS4C3 was indeed required for the transition between naïve and primed states, as mESCs differentiated into murine epiblast-like cells (Hirano et al., 2013). Such observations confirmed the importance of understanding rapid and subtle changes in HS fine structure during early differentiation events and tissue specification. Temporal and lineage-specific alterations in mESC HS structure can influence signaling pathways that mediate exit from pluripotency, highlighting HS as a key orchestrator of early developmental events.

Whilst many studies investigating the role of HS in pluripotent stem cell function have utilized mESCs due to their relative ease of culture and ability to readily produce genetic mutants, focus has begun to shift onto more relevant human ESCs (hESCs) and induced pluripotent stem cells (iPSCs). Gasimli and colleagues investigated changes in the structure of various GAGs, including HS, during the differentiation of hESCs toward mesodermal and endodermal lineages (Gasimli et al., 2014). Upon differentiation toward splanchnic mesoderm, several PG transcripts were markedly upregulated and an increase in HS sulfation was observed (predominantly *N*-sulfation). Differentiation toward an immature hepatocyte lineage yielded similar increases in HS sulfation, with an accentuation of *N*-sulfated, 6-*O*-sulfated HS (Gasimli et al., 2014). Such evidence highlights the potential importance of regulated variation in HSPG expression and HS fine structure during specification toward different germ layers and cell lineages. It also suggests a possibility that particular HSPG signatures, which predominate over others at distinct stages of lineage commitment, may be used to develop potential markers of differentiation. However, more evidence detailing chronological changes in HSPG composition and associated sequence motifs during lineage commitment is needed to improve an understanding of the relationship between the heparanome and differentiation status.

The use of hESCs has also expanded knowledge regarding the requirement of HS within the extracellular environment for guiding stem cell fate. A 2012 study indicated that matrix-bound HS is essential for the effective culture expansion and maintenance of pluripotency of hESCs (Stelling et al., 2012). Cells were cultured on a variety of substrates, including mouse embryonic fibroblasts (MEFs), ethanol-fixed MEFs, and MEFs devoid of HS. It was observed that hESCs maintained pluripotency marker gene expression when cultured on feeder layers presenting HS (both live and fixed). In contrast, HS-deficient layers were unable to support hESC attachment and growth (Stelling et al., 2012). Another group demonstrated that decellularized organ matrices were capable of influencing differentiation and lineage commitment of pluripotent stem cell-derived mesoderm progenitors through HSPG-bound tissue-specific factors (Ullah et al., 2020). Together, these data highlight that HS in *trans* within the surrounding matrix or culture substrata is crucial for the maintenance of homeostasis, as well as the regulation of ESC function.

### Neuroepithelial Cells

The significance of HSPGs in early central nervous system (CNS) development was established from studies where the absence of key biosynthetic enzymes resulted in pronounced developmental abnormalities (Poulain and Yost, 2015). Conditional knockout of *Ext1* in the nervous system of murine embryos gave rise to severe midbrain and cerebellar deformities, underdeveloped cerebral cortices and a lack of key neuronal tracts, leading to postnatal lethality (Inatani et al., 2003). Defects in forebrain development, cerebral hypoplasia as well as craniofacial malformations, such as hydrocephalus, have also been described with deficiencies in *Ndst1, Hs2st1*, and *Sulf2*, respectively (McLaughlin et al., 2003; Grobe et al., 2005; Kalus et al., 2009). Notably, these developmental phenotypes were observed to overlap extensively with those obtained by knocking out genes encoding key morphogens, such as *Fgf8*, *Wnt1*, and *Shh*, during embryogenesis (Thomas et al., 1991; Chiang et al., 1996; Meyers et al., 1998). This suggested an HS-dependency of these signaling factors in directing stem cell function for neural tube patterning.

Further inquiry revealed that HSPGs are essential prior to and during neurogenesis (Yamaguchi, 2001). Distinct spatiotemporal expression patterns of HS-binding FGFs have been observed to mediate the proliferation, survival and differentiation of neuroepithelial cells (NECs) as well as their radial glial cell (RGC) progeny (Murphy et al., 1990; Guillemot and Zimmer, 2011). For example, FGF2 and FGF8 support the expansion of NECs soon after specification in the neural tube, while the onset of FGF10 expression promotes the asymmetric division of mature NECs to form RGCs (Raballo et al., 2000; Storm et al., 2006; Sahara and O’Leary, 2009). Such nuanced FGF expression, FGF2 in particular, is accompanied by the presence of HSPGs as early as embryonic day 9 (E9) in murine neuroepithelial tissue (Nurcombe et al., 1993). Importantly, the functional properties of neuroepithelial HS were observed to change as embryogenesis progressed, in synchrony with changes in FGF expression. While HS derived from E9 tissue was able to bind FGF2 effectively in comparison to FGF1, a shift in binding affinity toward FGF1, rather than FGF2, was found in HS from E11. Such an alteration in binding affinity at E11 was attributed to compositional variations of HS, including longer chain length, greater number of sulfated domains and increased 6-*O*-sulfation in comparison to E9 chains (Brickman et al., 1998). Furthermore, a single core protein species was detected in both E9 and E11 neuroepithelial cultures, suggesting that HS chains on an individual PG may be rapidly modulated to complement, or even augment, the action of signaling molecules that regulate distinct NEC phenotypes (Nurcombe et al., 1993).

Several aspects of NEC behavior, including quiescence, responses to morphogen gradients, migration, self-renewal, and the generation of RGCs capable of differentiating, are regulated by HS-binding signaling factors. Moreover, distinct HSPGs appear to mediate such cellular events within the developing CNS. Perlecan has been identified to promote NSC proliferation by providing HS chains that function as co-receptors for FGF2 signaling, and also operate as an important component of the neural tube basement membrane (Joseph et al., 1996; Haubst et al., 2006; Girós et al., 2007). The presence of several membrane-bound syndecans has also been determined as important for neurogenesis. At E10, all 4 syndecans were found on the surface of NECs, with syndecan-1 mRNA expressed at the highest level (Ford-Perriss et al., 2003). Knockdown of syndecan-1 has been observed to attenuate canonical WNT signaling in NECs and RGCs, and severely diminish the proliferative capacity of these cells (Wang et al., 2012). This suggests that syndecan-1 normally functions to modulate proliferation-associated signaling cascades in early CNS development, much like syndecan-4, which has also been described to regulate proliferation during zebrafish neurogenesis (Luo et al., 2016). Additionally, syndecan-3 has been reported to facilitate neuroblast migration by triggering actin cytoskeletal changes. HS-dependent interactions with pleiotrophin and GDNF activate key intracellular signaling proteins, such as SRC kinase, and induce morphological changes required for cell motility (Raulo et al., 1994; Bespalov et al., 2011).

In addition to the syndecans, Ford-Perriss and colleagues also reported mRNA expression of most of the glypicans in E10 murine neuroepithelial tissue, with the exception of glypican-5 (Ford-Perriss et al., 2003). While knowledge regarding the exact functions of glypicans during neurogenesis remains limited, some studies have suggested roles in regulating stem cell proliferation, presumably via associations with FGFs. A loss of glypican-1 and corresponding FGF17 interactions in homozygous-null murine mutant embryos has been observed to hamper the proliferation of neuronal precursors and induce premature differentiation around E9, leading to a reduction in brain size (Jen et al., 2009). Maintenance of a proliferative stem cell phenotype has also been shown to depend on appropriate glypican-4 expression and putative FGF-binding. Glypican-4 has been observed to be persistently present in NECs within the ventricular zone but downregulated in their post-mitotic progeny (Hagihara et al., 2000). Although the mechanisms regulating such spatiotemporal variations in HSPG expression are yet to be elucidated, dynamic PG patterns seem to be hallmarks of neurogenesis, where distinct PG combinations function to fine-tune NEC responses at each stage of the developmental process. Such findings have led to the identification of particular HSPGs as potential biomarkers for neural lineage commitment, and have also suggested therapeutic targeting of these HSPGs for regulating neural stem cell function (Oikari et al., 2016; Yu et al., 2017).

## HSPGs as Regulators of Adult Stem Cell and Progenitor Functions

The role of HSPGs in adult stem cell (AdSC) populations is less well understood compared to ESCs and their progeny, primarily because such analysis is dependent on the ability to identify and purify tissue-specific stem cells. While some AdSC populations have been relatively easy to access for cell isolation, others remain elusive.

### Haematopoietic Stem Cells and Progenitors

Studies investigating the presence and role of polysaccharides in the bone marrow stroma gained momentum during the 1970s; a 1978 study demonstrated that GAGs or mucopolysaccharides could influence *in vitro* erythrocytic proliferation and differentiation (Ploemacher et al., 1978). Other investigations detected the presence of sulfated GAGs in the bone marrow stroma and suggested a role for stromal cells in producing them to support haematopoiesis (McCuskey and Meineke, 1973; Del Rosso et al., 1981). Simultaneous inquiry into the conditions required for long-term culture of HSCs and their progenitors (HPCs) revealed the dependence of these cells on adhesion with mesenchymal stromal cell feeder layers (Dexter et al., 1977; Reimann and Burger, 1979; Gartner and Kaplan, 1980; Bentley and Tralka, 1983), further endorsing the idea of stroma-derived HS as a putative mediator of cell–cell and cell–matrix interactions required for HSC survival and function.

The significance of stromal cell HS for *in vitro* haematopoiesis was confirmed when HS was found to be enriched in extracts of stromal feeder cells of long-term haematopoietic cultures and haematopoiesis-supportive bone marrow stromal cell lines (Gallagher et al., 1983; Wight et al., 1986; Kirby and Bentley, 1987). In a study by Gallagher et al. (1983), high proportions of disaccharide and tetrasaccharide species obtained by nitrous acid digestion of stromal HS demonstrated the presence of *N*-sulfate rich domains. Oligosaccharide mapping revealed that HS produced by cultured bone marrow stromal cells is enriched in highly sulfated disaccharides, indicating extensive protein-binding properties (Turnbull and Gallagher, 1988). Furthermore, work from our group has shown that in the presence of pro-haematopoietic cytokines, an exogenous 6-*O*-sulfate-rich bone marrow stromal cell-derived HS variant is capable of maintaining a subset of primitive HSCs during *ex vivo* expansion. These cells exhibit improved clonogenicity and an increased propensity to form erythroid and granulocyte progenitors, highlighting the capacity of 6-*O*-sulfate-rich HS fragments to enhance HSC properties (Bramono et al., 2011). Other studies have detailed the role of extracellular HS in inducing differentiation of naïve HPC-like cells. For example, HL-60 promyelocytic leukemia cells were observed to acquire a mature phenotype when grown on marrow stroma-derived matrix, which was abrogated upon heparinase treatment (Luikart et al., 1990). Moreover, the pro-differentiation effect was recapitulated when exogenous heparin was added to the cultures. The ability of HS/heparin in *trans* to guide HPC fate was similarly exhibited, when addition of HS to megakaryocyte progenitor cultures augmented megakaryocytopoiesis (Han et al., 1996).

To better understand the function of stromal HS during haematopoiesis, several studies investigated HS binding to bone marrow-relevant signaling molecules. Gordon and colleagues found that stroma-derived GAGs, of which HS was a major constituent, were capable of binding to granulocyte-macrophage colony stimulating factor (GM-CSF), while negligible binding was observed between liver-derived GAGs and GM-CSF (Gordon et al., 1987). They posited that this selective binding was due to stroma-specific structural features of GAGs that enabled compartmentalization and appropriate presentation of signaling molecules important for HPC function. In addition to GM-CSF, stroma-derived HS was also capable of binding interleukin (IL)-3, a key multilineage haematopoietic signaling factor (Roberts et al., 1988). Subsequently, various heparin/HS-binding proteins were found in the bone marrow stroma including GFs, chemokines as well as morphogenetic proteins such as BMPs, WNT, and SHH (reviewed in Papy-Garcia and Albanese, 2017).

In addition to its involvement in signaling events, HS produced by the bone marrow stroma was also found to be involved in the adhesion of HPCs and HSCs to stromal cells. Treating HPC-stromal cell cultures with sodium borohydride was observed to strip HS from cell-surface protein cores and destroy cell–cell binding activity (Siczkowski et al., 1992). De-sulfation of HSPGs as a result of sodium chlorate treatment was also found to decrease HPC adhesion to stromal feeder cells (Zweegman et al., 2004). Apart from adhesion, HSPGs in the marrow stroma are important participants in CXCL12/CXCR4-dependent cascades that orchestrate HPC migration, homing and retention, especially after transplantation. Netelenbos and colleagues conducted a study where subendothelial matrices produced by bone marrow endothelial cells were used to coat filters in a transwell migration assay using HPCs (Netelenbos et al., 2002). Cells were observed to migrate through the filters toward CXCL12 (stromal cell-derived factor-1; SDF-1)-enriched media, which did not occur in the absence of CXCL12. Importantly, HPC migration was significantly reduced when the matrix filter was treated with heparinases. This indicated that subendothelial ECM-associated HS was essential for trans-matrix migration in response to CXCL12 and is likely involved in CXCL12 presentation. A follow-up paper by the same group demonstrated that HS produced by bone marrow endothelial cells binds to cell surface CXCL12 in an autocrine manner, and assists in its interactions with CXCR4 on migrating HPCs (Netelenbos et al., 2003).

While the requirement of stromal HS in mediating HSC and HPC function has been well established, the significance of specific sequence motifs and particular PG core proteins remains to be understood. In-depth compositional analysis of stroma-derived HS coupled with gene expression profiling of HSPG core proteins in stromal cells may provide preliminary insights. Nonetheless, studies exploring the significance of HS in HSC and HPC function has progressed at a rapid pace, whereas evidence of the roles of HS in other marrow resident progenitor functions has unfolded more slowly. An important factor that has facilitated thorough investigations of HSC properties is a well-defined, robust *in vitro* culture system. This highlights the need for similar approaches to be adopted to further our understanding of other tissue-specific stem cell types that may be less amenable to *ex vivo* expansion.

### Mesenchymal Stem Cells

Mesenchymal stem cells (MSCs) were initially described as marrow-derived proliferative, colony-forming and plastic adherent cells (Friedenstein et al., 1974). Subsequent studies led by Caplan’s group to better characterize these cells led to the discovery of their multilineage potency: they were able to form osteoblasts, chondrocytes and adipocytes *in vitro* and when transplanted *in vivo* (Osdoby and Caplan, 1976; Caplan, 1991). Although the *in situ* identity and function of MSCs has remained controversial, these cells continue to be widely studied and are valuable tools for use in cell therapy applications, especially by virtue of their immunomodulatory secretome (Weiss and Dahlke, 2019).

An important challenge for the successful clinical use of MSCs is their inherent heterogeneity, particularly after culture expansion. MSCs have been reported to show variability in both surface marker expression and function, including biased differentiation potential and clonogenic capacity, depending on their tissue of origin and/or culture conditions (da Silva Meirelles et al., 2006; Traktuev et al., 2008; Hagmann et al., 2013; Holley et al., 2015; Elabd et al., 2018). While transcriptomic approaches have dominated the toolkit for MSC analysis, glycomic analysis and GAG profiling of MSCs are yet to be adopted for more comprehensive characterization. As discussed earlier (see section “Heterogeneity in HSPG Composition and Localization Can Influence Stem Cell Fate”), heterogeneity in HS composition can influence stem cell fate decisions. Owing to the significant tissue-dependent differences observed in MSC functional properties, it is highly likely that these are accompanied by distinct tissue-specific HS signatures. Therefore, we deem it prudent to investigate the status of HSPGs, as well as other GAGs/PGs, in MSCs.

Together with transcriptomic analysis, glycomic data will allow for a better understanding of the mechanisms related to the functional heterogeneity of these cells. Furthermore, knowledge regarding the roles of particular HS signatures in mediating MSC properties will allow for the synthesis of desirable HS adjuvants to culture media (see section “Exploiting the HSPG-Stem Cell Relationship for Therapeutic Use: HS as an Adjuvant for the Expansion of Potent MSCs”). This approach may permit the development of improved expansion regimens, wherein tailored HS oligosaccharides are capable of maintaining reservoirs of appropriate GFs and other signaling molecules in the ECM for better control of MSC fate *in vitro.*

### Osteogenic and Chondrogenic Progenitors

Osteogenic and chondrogenic differentiation have been widely studied *in vitro*. Both trajectories involve drastic changes in the composition of the ECM, with the laying down of a mineralized matrix being a hallmark of osteogenesis (Assis-Ribas et al., 2018). Importantly, alterations in the expression of genes encoding PG core proteins and HS biosynthetic machinery have been reported during osteogenesis (Haupt et al., 2009). Pre-osteoblasts cultured in osteogenic media were observed to increase the mRNA and protein levels of several PG core proteins as they matured. While glypican-3 displayed the most significant increase, followed by all 4 syndecans, glypican-2 and glypican-4 showed moderate increases. There were also variations in gene expression levels of some biosynthetic enzymes: a decrease in *Ext1/2* gene expression was noted, with contrasting increases in *Ndst1/2*, *Hs2st1*, and *Hs6st1* as differentiation progressed.

A study from Zhao and colleagues also reported an upregulation of 6OST3 at the mRNA and protein levels as MSCs underwent calcification (Zhao et al., 2015). Collectively, data from these studies indicate that as pre-osteoblasts or MSCs progress through osteogenesis, HS chain initiation may decrease while overall chain sulfation may be increased. Moreover, siRNA knockdown of glypican-3 or 6OST3 was found to decrease differentiation down the osteogenic lineage (Haupt et al., 2009; Zhao et al., 2015), suggesting the necessity of unique HSPG motifs for progression through the lineage commitment process. Further investigations into the functional features of unique HS/heparin signatures in osteogenesis have demonstrated distinct binding properties for proteins that mediate important pro-differentiation signaling cascades. For example, bone marrow-derived HS is able to bind with high affinity to, and enhance the osteoinductivity of BMP2 (Bramono et al., 2012). In another study, highly sulfated heparin was found to bind WNT3A with high affinity and promote the osteogenic activity of pre-osteoblasts (Ling et al., 2010). To improve our understanding of how the heparanome may be altered during osteogenesis and the resulting functional effects this may have on stem cell differentiation, additional studies may be undertaken where HS pools are extracted from cultures at various stages of the osteogenic process. Subsequent HS disaccharide isolation and characterization would provide information regarding the compositional features of HS during differentiation, and also offer evidence to complement existing gene expression data for biosynthetic enzymes.

The role of HS as an important regulator of chondrogenesis was first demonstrated in 1987 using micromass cultures of chick limb bud mesenchyme (San Antonio et al., 1987). Addition of either exogenous HS or heparin was found to enhance chondrogenesis and cartilage nodule formation in a dose-dependent manner. The involvement of HSPG core proteins in chondrogenesis has also been demonstrated *in vitro* (Gomes et al., 2004). The ability of perlecan to induce chondrogenesis was tested by plating MSCs on surfaces coated with intact perlecan or recombinant perlecan domain 1. Plated cells were found to attach and aggregate into dense cell condensations, and proceed to express chondrogenic differentiation markers such as collagen type II and aggrecan. This phenotype was not obtained with cells plated with other PGs or perlecan domains, suggesting that only certain HSPGs possess structural features that support chondrogenesis. Other groups have further explored the role of HS in chondrogenesis primarily by assessing the effects of exogenous HS on chick limb mesenchymal cells (LMCs) or MSC cultures. Addition of HS or BMP2 alone to LMC cultures led to only a moderate improvement of Alcian blue staining (Fisher et al., 2006). However, the addition of HS and BMP2 together significantly accentuated staining compared to individual treatments. The presence of HS was able to potentiate the activity of BMP2, as indicated by increased phosphorylation of SMAD proteins, and lowered the concentration required to stimulate chondrogenesis to an equal or even greater extent as compared to high concentrations of BMP2 alone. Similarly, combined exposure of HS and TGF-β3 resulted in higher cartilage-specific gene expression levels and even induced remodeling of the surrounding microenvironment by increasing cartilage matrix protein production compared to TGF-β3-only or HS-only treatments (Chen et al., 2016).

While the significance of HSPGs in mediating chondrogenesis has been documented mainly through examining the effects of exogenous HSPGs on differentiation, the heparanome status of the stem cells themselves during chondrogenic lineage commitment is unknown. Gene expression analysis of biosynthetic enzymes coupled with glycomic profiling of cultures undergoing chondrogenesis would strengthen our knowledge of how HSPGs may influence this differentiation process. Furthermore, such data would provide a basis for subsequent mechanistic studies that probe into the functional attributes of the heparanome as chondrogenesis ensues.

### Muscle Satellite Cells and Myoblasts

Satellite cells (MuSCs) are stem cells within skeletal muscle that are normally quiescent within a specialized niche located between the plasma membrane and basement membrane of myofibres (Mauro, 1961). They exit quiescence, resume cell cycle activity and acquire a myoblast identity for muscle regeneration, in response to triggers such as exercise or injury. A fine balance of signaling events is essential for an appropriate MuSC response. Of the many families of signaling factors, FGFs emerged as key participants in mediating MuSC activity (Pawlikowski et al., 2017). Studies in the 1980s showed that exposure of muscle progenitors to FGF1 or FGF2 resulted in strong mitogenic effects and concomitant repression of terminal differentiation (Lathrop et al., 1985; Clegg et al., 1987). FGF deprivation of cells that were initially exposed resulted in withdrawal from the cell cycle and simultaneous expression of myosin heavy chain. The indication of FGFs as inhibitors of myogenic differentiation was further corroborated when it was shown that exposure of myoblasts to FGF also inhibited myogenin expression (Brunetti and Goldfine, 1990). Not long after, the integral role of HS, and their 6-*O*-sulfated residues in particular, in facilitating FGF signaling in MuSCs became evident. Rapraeger and colleagues demonstrated that treatment of MM14 cultures with either sodium chlorate or heparinase abolished FGF signaling (Rapraeger et al., 1991). Cells ceased proliferation and stained positive for myosin heavy chain, recapitulating results obtained upon FGF deprivation (Lathrop et al., 1985; Clegg et al., 1987). In a follow up study, the group reported that the addition of heparin to chlorate-treated cultures rescued FGF signaling, further highlighting the role of HS in mediating FGF-dependent repression of myogenic differentiation (Olwin and Rapraeger, 1992).

The use of the MM14 culture system has provided information on another key feature of myogenesis, which is a change in the expression of myoblast HSPG core proteins. Analysis of MM14 PGs showed a decrease in syndecan expression in differentiated cells compared to proliferative counterparts (Olwin and Rapraeger, 1992). A decrease in syndecan-3 expression along with perlecan, at both protein and mRNA levels, was also observed as C2C12 myoblasts differentiated into myotubes (Larraín et al., 1997; Fuentealba et al., 1999). Inhibition or genetic ablation of syndecan-3 expression in myoblasts or MuSCs drastically decreases sensitivity to FGF2-dependent inhibition of myogenesis, and also precludes signaling via the Notch pathway (Fuentealba et al., 1999; Pisconti et al., 2010). As a result, cells undergo reduced self-renewal and precocious acquisition of a differentiated phenotype, which can be reversed by exogenous heparin. In contrast to syndecan and perlecan, glypican-1 expression has been observed to be maintained both on the cell surface and as a secreted form in the ECM throughout the myogenic process (Campos et al., 1993; Brandan et al., 1996). Localization of glypican-1 in lipid rafts at the cell membrane was found to be an important mechanism that sequesters FGF2, prevents interactions with FGFRs and inhibits myoblast differentiation. A deficiency in glypican-1 was unable to restrict FGF:FGFR interactions and led to defective differentiation of C2C12 cells (Gutierrez and Brandan, 2010). Collectively, these results indicate that intricate spatiotemporal variation and control of HSPG expression *in vitro* is pivotal in modulating FGF signaling for myogenic activity.

Further investigations of the roles of HSPGs in myogenesis have extended such *in vitro* findings and shown that heterogeneity in core protein expression *in vivo* is crucial during muscle development and in adult skeletal muscle. While syndecans-1, -3, and -4 were found to be enriched in developing mouse skeletal muscle tissue, only syndecan-3 and syndecan-4 expression persisted in adult muscle, colocalized with FGFR1 (Cornelison et al., 2001; Olguin and Brandan, 2001). Cornelison and colleagues observed that the expression of both these FGFR-associated syndecans overlapped with c-MET, a key MuSC marker, suggesting a role for HSPG-mediated signaling in MuSC function *in vivo*. Moreover, treatment of adult myofibre explant cultures with sodium chlorate led to a significant decrease in MuSC numbers, and an associated increase in the percentage of cells expressing MyoD (Cornelison et al., 2001). Thus, a switch from MuSC proliferation to differentiation could be triggered in response to disruption of HS-dependent FGF signaling in MuSCs. The significance of specific patterns of HSPG expression in adult skeletal muscle was further supported by a study conducted by Casar and colleagues. They detected widespread HSPG expression by immunostaining [using the 3G10 monoclonal antibody (David et al., 1992)] in the myofibres of healthy mouse muscle (Casar et al., 2004). Following injury, an increased 3G10 signal was detected in the regenerating myotubes. Subsequent analysis of upregulated HSPGs identified syndecan-3, syndecan-4, glypican, and perlecan, with syndecan-3 upregulated the earliest in regenerating structures. In comparison, dysregulated HSPG expression, as in the case of syndecan-3^–/–^ and syndecan-4^–/–^ mice, severely impacted the ability to maintain homeostasis in the myogenic tissue and activate regenerative programs upon injury (Cornelison et al., 2004).

Apart from dynamic PG core protein expression patterns, variability in HS biosynthetic enzyme expression has also been identified in MuSCs. Langsdorf and colleagues found that only SULF1 was expressed, both at protein and mRNA levels, in quiescent mouse MuSCs. However, following muscle injury, a shift in *Sulf* expression was observed such that both SULF1 and SULF2 were detected in *Pax7*-positive MuSCs. To investigate the role of the SULF enzymes in myofibre regeneration, the group went on to characterize *Sulf* double mutant mice (Langsdorf et al., 2007; Tran et al., 2012). In the absence of *Sulf1* and *Sulf2*, the animals displayed an acutely delayed and compromised ability to regenerate muscle. GAG disaccharide analysis revealed that the lack of SULF enzymes led to a significant increase in 6-*O*-sulfation, with a notable rise in trisulfated disaccharide levels. Such an overtly sulfated HS signature was associated with an overactivation of the FGF pathway as well as an elevation in non-canonical WNT signaling, leading to an inhibition of differentiation and myoblast fusion (Langsdorf et al., 2007; Tran et al., 2012). These results identified SULF proteins as repressors of inhibitory FGF signaling in activated MuSCs and important gatekeepers of HS composition.

Although specific patterns of PG and biosynthetic enzyme expression seem to be important for myogenic activity during development and regeneration, the significance of specific HS domain structures and sulfation patterns in mediating these processes is largely unknown. Future studies focused on glycomic characterization are required for a better mechanistic understanding of the functions of HS in regulating myoblast or MuSC fate.

### Skin Stem Cells and Progenitors

Homeostasis within the skin layers and its appendages is maintained by several distinct stem cell and progenitor populations. The epidermal basal layer consists of rapidly diving stem cell progenitors (transit-amplifying cells) that work to continually replenish the epidermal layers (Potten and Morris, 1988; Fuchs and Raghavan, 2002). In response to appropriate triggers, these cells lose their attachment, both to one another as well as the underlying basement membrane (BM), and differentiate into keratinocytes (KCs) destined for stratification (Barrandon and Green, 1987; Blanpain and Fuchs, 2009). The involvement of HS and its PGs in the functional fidelity of KCs and the structural integrity of the skin BM has been an important area of research.

The role of HSPGs as key regulators of signaling is conserved in epidermal progenitors, as in the case of other previously discussed AdSC types. Several studies have highlighted the necessity of HS-binding GFs, including FGFs, GM-CSF and TGF-β, for the control of basal layer progenitor proliferation as well as differentiation (Mansbridge and Hanawalt, 1988; Finch et al., 1989; Szabowski et al., 2000; Zhu et al., 2014). The expression of HSPG core proteins has also been well established within the epidermis as well as along the entire length of the dermo-epidermal junction in the BM (Hassell et al., 1980; Caughman et al., 1987; Horiguchi et al., 1989). Importantly, the patterns of core protein expression within the epidermis are dynamic and have been found to change as KCs progress through terminal differentiation. Syndecan-1 expression was reported as modest in the basal layer, enriched in the suprabasal layer, and absent in superficial layers (Sanderson et al., 1992). This trend in syndecan-1 expression was recapitulated when stratification of KC monolayers was induced *in vitro*. The purified syndecan-1 from stratified samples was found to be more abundant than in monolayer controls and also had a lower molecular mass, which was attributed to differences in the composition of HS chains (Sanderson et al., 1992).

Unlike the syndecans, the expression patterns of glypicans are less well known in the epidermis. A study from 2006 utilized an immunohistochemical staining approach and detected diffused glypican-1 expression throughout the epidermis, while glypican-3 appeared restricted to the basal layer (Patterson et al., 2008). These results collectively indicate that unique profiles of HSPGs at different stages of KC differentiation serve specific functions in mediating cell fate decisions. In this regard, Sanderson et al. (1992) suggested that changes in HSPG composition are essential to facilitate the drastic alterations in KC adhesive properties required for detachment from the basal layer and subsequent migration. Likewise, the presence of HSPGs, such as perlecan, within the ECM has been found to heavily influence KC migration and organization. In a study by Sher et al. (2006), perlecan was detected in all epidermal layers apart from the stratum corneum when KCs were seeded on dermal equivalents. However, upon transfecting a perlecan antisense construct into KCs, differentiation was significantly altered. The cells formed an aberrantly organized epidermis that was only 1–2 layers thick. Notably, exogenous perlecan addition rescued the ability of KCs to form a well-differentiation, stratified epidermis.

Apart from the epidermis, HSPGs, syndecans in particular, have been identified in stem cell-rich hair follicles (HFs) (Couchman, 1993). To elucidate the role of HS in the HF, Coulson-Thomas et al. (2014) characterized a transgenic mouse model that was devoid of *Ext1* only in the outer root sheath (ORS), inner root sheath (IRS) and hair shaft. They observed a significantly increased number of HFs in transgenic mice compared to littermate controls. These HFs were observed to be arrested at anagen, unable to transition into catagen and telogen. Subsequent analysis revealed that cells within these HFs had impaired differentiation potential, due to uncontrolled signaling involving SHH. Thus, in the absence of HS, control of the intricate molecular networks that preside over stem cell fate decisions during HF cycling and homeostasis is severely perturbed. Widespread HSPG expression has also been observed in dermal papillae (DP), important stem cell-containing signaling centers at the base of HFs that regulate epithelial stem cells and keratinocytes for HF cycling (Driskell et al., 2011; Morgan, 2014). Notably, HSPG expression patterns have been found to be spatiotemporally variable in the DP matrix and the adjacent ORS (contiguous with the epidermal BM) throughout HF cycle stages, except for telogen (Couchman, 1986; Westgate et al., 1991; Couchman, 1993). While perlecan expression persisted in both the BM and DP, syndecans localized at different positions during the HF cycle (Kaplan and Holbrook, 1994). During anagen, syndecan-1 is strongly detected in the ORS of the HF and to a lesser extent in DP (Malgouries et al., 2008). As follicles proceed through catagen, syndecan-1 levels diminish in the ORS, below that within the DP (Bayer-Garner et al., 2002).

Stem cell populations within the epidermis, HF and DP appear to share a commonality in that distinct PG core protein expression patterns appear to correlate with specific stem cell responses and fate decisions. However, the role of HS chains attached to these proteins and their functional significance is currently unknown. The extraction of HS from epidermal tissue and subsequent structural analysis may provide preliminary insights into the overall status of the epidermal heparanome, and may also help identify sequence motifs that impart protein-binding properties.

### Cancer Stem Cells

Dysregulated HSPG biosynthesis and aberrant chain modifications have been documented in several solid tumors and hematological malignancies. Changes in HS sulfation patterns due to alterations in expression of the sulphotransferases and SULF enzymes, coupled with increased cleavage by HPSE, have long been implicated in key tumorigenic events (Vlodavsky et al., 2007; Rosen and Lemjabbar-Alaoui, 2010; Hammond et al., 2014; Nagarajan et al., 2018). Increased localization of HS binding signaling factors and ECM remodeling events (such as breakdown of HSPGs in the basement membrane) are known to underpin tumor proliferation, angiogenesis and metastasis (Knelson et al., 2014). Research over the last decade has also uncovered vital roles for HSPGs in mediating cancer stem cell (CSC) function.

CSCs have been described to fuel tumor growth and heterogeneity due to their highly proliferative, phenotypically plastic, and treatment-resistant nature (Plaks et al., 2015; Batlle and Clevers, 2017). Several HSPG signatures have been identified to promote CSC survival and oncogenic properties. For example, upon profiling triple negative inflammatory breast CSCs, Ibrahim, and colleagues found significantly high levels of syndecan-1 mRNA and protein levels (Ibrahim et al., 2017). Notably, siRNA knockdown of syndecan-1 was observed to directly affect CSC survival and decrease the pool of available cells. These knockdown cells were unable to efficiently form colonies and spheroids, highlighting a perturbation in their self-renewal capacity. Such a dependency on syndecan-1 for maintaining oncogenic CSC activity was also observed in murine mammary glands (Liu et al., 2004). Overexpression of *Wnt1* failed to trigger the accumulation of mammary CSCs and progenitors for oncogenic transformation in syndecan-1-null mice, whereas an enrichment in the progenitor population and tumor initiation were induced in syndecan-1 expressing counterparts. A follow up study showed that the tumor resistance conferred by the absence of syndecan-1 extended beyond the mammary glands in null mice and was a multi-organ effect (McDermott et al., 2007). While this evidence clearly indicates an involvement of syndecan-1 in augmenting the tumorigenic properties of CSCs, it is unclear whether this phenomenon relates to the properties of the HS chains they carry. Further investigation is required to establish if syndecan-1-dependent enhancement of CSC activity is an HS-independent feature or not.

Although syndecan-1 may be important for tumorigenesis in certain tumor types, patterns of HSPG expression in CSC populations appear to be contextual and tissue-specific. For example, decreases in syndecan-1 expression have been observed to correlate with epithelial-mesenchymal transitions (EMT) and poorly differentiated phenotypes in colon cancer (Hashimoto et al., 2008). To understand the significance of syndecan-1 in colon CSCs, Kumar Katakam et al. (2020) performed an siRNA knockdown study. Silenced cells showed enhanced expression of stemness markers, such as *SOX2* and *NANOG*, an accentuated ability to self-renew and a greater tendency to undergo EMT compared to control cells. Furthermore, it was observed that dampened syndecan-1 levels in the knockdown cells were accompanied by a concomitant rise in WNT signaling and expression of downstream transcription factors of the TCF/LEF family, boosting the CSC phenotype. Attenuated HSPG expression in colon CSCs was thus sufficient to induce pro-oncogenic features and trigger associated signaling events. In a follow-up study, the group also found that HPSE expression was elevated in syndecan-1 depleted colon cancer cells (Katakam et al., 2020). These cells exhibited enhanced stem cell properties including sphere formation capacity and strong expression of stemness markers. While such observations suggest the involvement of HS remodeling mechanisms in tumor progression, accompanying analyses at the level of HS compositional characterization and associated bioactivity will be essential to confirm this, and to discern whether particular glycan signatures are associated with enhanced CSC function. Apart from the syndecans, variations in CSC glypican expression have also been reported. Enriched glypican-4 expression has been identified in chemotherapy-resistant pancreatic CSCs (Cao et al., 2018). Knockdown of glypican-4 resulted in a dramatic decrease in stemness marker expression (such as *OCT4*, *SOX2*, and *NANOG*) in these cells and increased their vulnerability to 5-fluorouracil, a chemotherapeutic agent. Moreover, glypican-4 knockdown cells exhibited suppressed WNT signaling and a decreased level of nuclear β-catenin. This highlights a dependence on the expression of particular HSPGs for the progression of important signaling cascades that accentuate stemness features in CSCs.

In addition to the HSPG core proteins, the significance of unique HS fragments in mediating CSC responses has also emerged as an important area of research, mainly through investigating the effects of exogenous HS on CSC properties. Patel et al. (2016) adopted a screening strategy to test the effects of treating CSCs with a library of heparin/HS oligosaccharides of varying chain lengths. These oligosaccharides shared a common repeating disaccharide structure comprising a 2-*O*-sulfated iduronic acid residue linked to a glucosamine residue with sulfate moieties at the C6 and *N* positions (i.e., IdoA2S-GlcNS6S). The group discovered a chain length-dependent inhibition of self-renewal in CSCs across a variety of tumor cell lines. Spheroid growth was inhibited upon exposure to HS chains from dp6 to dp12, while longer and shorter chains were ineffective. Importantly, the dp6 oligosaccharide was the most potent and treatment was found to induce a decrease in the expression of CSC markers *CD44* and *LGRF5*. The group found that these effects on CSCs were brought about by a dp6-dependent activation of a specific isoform of p38, a stress-activated mitogen activated protein kinase (MAPK) with tumor suppressive functions. Although the mechanistic details of this interaction are yet to be fully elucidated and the functional properties of the HS fragments require validation, the results from this study offer important insights into the roles of HS oligosaccharides as anti-cancer therapy agents, depending on their compositional signatures.

Examining how chain attributes, such as length, domain organization, sulfate modifications and protein binding sites, may be tailored for the synthesis of HS oligosaccharides with desirable therapeutic properties holds immense promise in expanding the scope of glycotherapeutics. Moreover, modulating the expression levels and localization of HSPG core proteins, and utilizing methods to enhance or inhibit HS function may be adopted as potential approaches to regulate the heparanome of CSCs and consequently, ameliorate their aberrant behavior.

## Experimental Methods to Strengthen Our Understanding of the HSPG-Stem Cell Relationship

Current knowledge regarding the specific compositional and associated functional features of HSPGs in mediating AdSC fate is limited, with unaddressed gaps in mechanistic understanding and restricted clinical relevance. To acquire a holistic appreciation of the HSPG-stem cell relationship, future studies may adopt a multi-omics approach that examines the heparanome of different stem cell types using a combination of glycomic, genomic, chemical, and proteomic methods. Thorough HS compositional profiling would be useful for uncovering distinct glycomic signatures that may predominate during differentiation down a particular lineage. Employing liquid chromatography-tandem mass spectrometry (LC-MS/MS) would allow for the necessary identification of fine structural features of HS and associated temporal changes, with a high level of sensitivity and accuracy (Zaia, 2013). Additionally, perturbation of HS structure and subsequent loss-of-function effects in differentiating stem cells may be investigated to identify the required structural nuances and roles of HSPGs at distinct time-points during lineage commitment.

Apart from traditional genetic manipulation approaches, gene editing through drug-inducible CRISPR/Cas9 systems may be utilized to knockout key HS biosynthetic and regulatory enzymes or PG core proteins (Cao et al., 2016; Sun N. et al., 2019). The loss of such key HSPG-related proteins at specific stages of differentiation will provide insights into the relationship between the HSPG profiles of progenitors or stem cells and their differentiation status. A range of chemical methods have also been developed to inhibit HS function *in vitro*, including the use of sodium chlorate, xylosides, and surfen (Humphries and Silbert, 1988; Garud et al., 2008; Weiss et al., 2015). Xylosides serve as primers for HS chain synthesis, while fluoroxylosides prevent chain elongation and effectively inhibit HS and CS/DS biosynthesis (Garud et al., 2008). In a 2018 study, Huang and colleagues investigated effects of the heparin/heparan sulfate antagonist surfen (bis-2-methyl-4-amino-quinolyl-6-carbamide) (Weiss et al., 2015) on HS function in *Oct4*-GFP and *Sox1*-GFP mESC reporter lines (Huang et al., 2018). Treated cells showed persistent pluripotency and an inability to differentiate, iterating a requirement of HS in mediating lineage commitment decisions. Moreover, this effect was reversible through the removal of surfen, suggesting that this molecule could prove useful for the temporal inhibition of HS activity throughout various stages of differentiation *in vitro* (Huang et al., 2018).

Other than chemical inhibition, the most widespread method to perturb HS function is the use of heparinases isolated from bacteria (*Flavobacterium heparinum*). When all three enzymes are used together, HS chains are cleaved into constituent disaccharides. Whilst this approach is simple to execute in cell culture models, the rapid turnover of cell surface HS allows only for a limited period to conduct subsequent live cell experiments. Alternatively, heparinase digestion may be carried out prior to fixation of cells, followed by immunostaining and flow cytometry analysis (Ayerst et al., 2017). HS participation as a signaling co-receptor can also be inhibited through a variety of peptide or protein-based methods. The HS/heparin-binding domain of HS binding proteins may be engineered, such that GAG-protein interactions are precluded. A non-HS binding variant of BMP2 has been generated in this manner, through the replacement of the first 12 of the 17 N-terminal amino acids which constitute the heparin-binding domain (Ruppert et al., 1996; Kuo et al., 2010). Alternatively, HS-protein interactions can be hindered by ligand binding competition. For example, synthetic heparin-binding peptides competed with and prevented the attachment of cytomegalovirus envelop proteins to cell surface HS in fibroblasts (Dogra et al., 2015). Another approach involves the use of soluble decoy FGFR fusion proteins, which compete with cell surface FGFR for FGF binding and affect ternary complex formation with HS (Harding et al., 2013; Li et al., 2014). HS/heparin binding to protein partners and signaling complex formation may also be intercepted by the use of custom designed antibodies that mask the GAG binding site on the protein. Our lab has previously developed an antibody (IMB-R1) that was able to bind to FGFR1 and prevent FGFR1-heparin interactions (Ling et al., 2015).

## Exploiting the HSPG-Stem Cell Relationship for Therapeutic Use: HS as an Adjuvant for the Expansion of Potent MSCs

The critical roles HS plays in mediating stem cell function *in vitro* and *in vivo* makes it an ideal candidate for use as an adjuvant in cell therapy and associated culture expansion strategies. Our own work has focused on enhancing the expansion of potent human MSCs (hMSCs) by using exogenous HS as a supplement to culture media throughout expansion. Initial studies using rat MSCs revealed that the addition of exogenous HS resulted in enhanced proliferation and enhanced osteogenic differentiation (Dombrowski et al., 2009). Importantly, this effect was potentiated through FGFR1, a mechanism that has since become an extensive focus in our studies. In 2012, we sought to understand the effects of adding exogenous HS (HS2, a murine embryonic forebrain-derived variant that displays enhanced binding affinity toward FGF2) to hMSCs during *in vitro* culture (Helledie et al., 2012). As with rat MSCs, addition of exogenous HS augmented hMSC proliferation, especially of subpopulations with long telomeres and high expression of multipotency markers as well as the cell surface marker STRO-1. When HS2-treated hMSCs were assessed in an animal model of a critical-sized bone defect, bone regeneration was enhanced over hMSCs which were not pre-cultured with HS2. This gave us some indication that HS, specifically the HS2 variant, could contribute to the selection and expansion of a more potent sub-population of hMSCs.

Due to the difficulties of isolating sufficient HS2 from developing mouse brains for extensive analyses, we went on to develop an affinity isolation platform that utilizes the heparin-binding domain (HBD) of proteins. HBDs were peptide synthesized and bound to streptavidin-functionalised resin through a terminal biotin molecule (Murali et al., 2013; Wang et al., 2014; Wijesinghe et al., 2017). Using such methods, we developed a peptide modeled on an HBD from within FGF2. We then fractionated commercial porcine intestinal mucosal HS over the column. The retained material, termed “HS8,” was subjected to comprehensive testing against hMSCs (Wijesinghe et al., 2017). Addition of HS8 to culture media enhanced the proliferation and colony-forming efficiency of hMSCs, much like the effects observed after the addition of HS2. HS8 enhanced the stability of FGF2 in media and led to an accumulation over time, resulting in prolonged FGFR1 and ERK1/2 phosphorylation in hMSCs treated with sub-optimal concentrations of FGF2. The affinity isolation methodology demonstrated our ability to effectively scale production of a desired HS variant, yielding larger quantities suitable for more extensive biochemical and cellular analysis, including larger animal studies. HS variants isolated in this manner proved to be amiable to gamma irradiation, without a loss of structure or function (Smith et al., 2018). This technique could be used to sterilize potential HS-based cell media supplements prior to use in stem cell expansion. We next employed a microbioreactor array to further elucidate the mechanism by which HS8 functions in the *in vitro* hMSC microenvironment (Titmarsh et al., 2017). Using microfluidics, various concentrations of HS8 and FGF2 were mixed and applied to hMSCs under constant flow across sequential flow cells. The addition of HS8 not only increased production of endogenous FGF2, but also facilitated release of FGF2 from the cell surface (and likely ECM), resulting in perfusion of released FGF2 into downstream flow cells. HS8 also sustained FGF2 availability over time, an effect we have observed in previous studies.

Our most recent work explored the therapeutic potential of clinically sourced hMSCs isolated from fresh bone marrow aspirates (Ling et al., 2020). Extensive analysis revealed that HS supplementation of culture media increased hMSC proliferation, telomere length, expression of desirable cell surface markers and multipotency. We hypothesize that this is a result of the expansion of a sub-population of MSCs that display a more “naïve” phenotype. MSCs were subsequently expanded in media with or without HS8 and used in two animal models of a knee osteochondral defect (rat and pig). In the rat model, MSCs cultured with HS8 demonstrated enhanced defect healing over the control groups (empty, carrier, MSCs cultured without HS8), including increased type II collagen and GAG deposition within the wound site. Using a large animal model of an osteochondral defect, the micro pig, we found that animals treated with cells cultured in HS8 showed a marked improvement in wound healing, increased collagen II and GAG deposition, and improved mechanical properties at 4 and 8 months post-surgery. Throughout these studies, there was clear indication that supplementation of HS to the *in vitro* MSC microenvironment leads to an expansion of an MSC subpopulation with increased potency. HS supports this potent MSC cell type at least in part through its influence as a co-receptor in the FGF2:FGFR1 signaling cascade. Indeed, the rapid expansion of MSCs to desirable quantities for cell therapies often utilizes large quantities of FGF2 and other recombinant factors, many of which are heparin-binding. For this reason, the development of HS variants for the purpose of stem cell expansion could yield greater numbers of more potent cells by promoting the secretion of autocrine factors, or by increasing the stability of decreased concentrations of the exogenous factors ordinarily supplemented into the culture media.

Apart from media supplementation, HS may be introduced into cell cultures in other formats. In a 2019 study, Treiger and colleagues developed a novel heparinoid-bovine serum albumin (BSA) bioconjugate, wherein a copper-free click reaction was used to conjugate various heparin derivatives to cyclooctyne-functionalized BSA (Trieger et al., 2019). These constructs were passively adsorbed onto tissue culture plastic surfaces and served as ECM PGs. They were found to variably sequester FGF2 based on their sulfation patterns, resulting in increased proliferation of cultured hMSCs. One benefit of such reactions and methodologies is the ability to use a generic anchoring molecule (in this case, BSA) for surface coating, whilst a library of different functional moieties may be generated and employed depending upon the required use. Surface coatings offer potential advantages over traditional supplementation in solution: firstly, a much lower quantity of HS is required to achieve a maximum surface coating. Secondly, surface coatings more effectively recapitulate the extracellular environment of a typical stem cell, as HSPGs are either found contained within the ECM, on the cell surface or immediately adjacent to it, not as free-floating HS chains in solution. Finally, the large-scale culture of hMSCs requires the use of large bioreactors, utilizing microcarriers to which hMSCs may bind and proliferate in suspension. The use of HS-coated microcarriers should greatly reduce the amounts of HS required for such culture systems over HS supplementation into the bulk media. The last of these points is yet to be investigated but could provide useful insights into the importance of HS localization during stem cell expansion in artificial environments.

## Concluding Remarks

HSPGs have emerged as key mediators of stem cell function, essential for the regulation of development, homeostasis and regeneration. Early evidence from ESC studies highlighted the significance of HSPGs and their biosynthetic machinery in mediating key lineage commitment and cell fate decisions. Recent observations have indicated conservation in HSPG function and compositional regulation across AdSC types, as well as perturbation in analogous mechanisms in CSC populations. The use of HS variants as media adjuvants offers an encouraging avenue for the development of customized media formulations for bioprocessing cells suitable for clinical application. However, further investigations are required to unravel the importance of particular HSPG compositional characteristics and associated functional features in guiding the behavior of various stem cell types, especially prior to clinical adoption.

It is worth noting that in our own experience, the structure and composition of HS varies widely depending upon the source, with highly sulfated HS variants more akin to heparin. We and others have previously discussed the negative impact of long-term culture supplementation with heparin has on the molecular phenotype of MSCs (Hemeda et al., 2013; Ling et al., 2016), in addition to the negative impact heparin has on GDF5 signaling (Ayerst et al., 2017). Yet, we do not observe such phenomena with HS, a molecule which relies on smaller but distinctly heparin-like NS domains to facilitate interactions with ligands and receptors. These subtle distinctions in structure, which yield significant differences in subsequent biological events, require more exhaustive examination. Rigorous inquiry into the biochemistry of HSPGs will accelerate their development as tools and pharmacological agents to modulate stem cell responses *in vitro* or *in vivo*, and is a necessary step to be undertaken if HS is to see widespread therapeutic application. Despite this, the amalgamation of glycotherapeutics and stem cell therapy holds considerable promise in bringing forth novel strategies for regenerative medicine.

## Author Contributions

MR and RS conceptualized, drafted and edited the manuscript. VN contributed to editing the manuscript. SC was invited by the journal to provide the review, revised the manuscript and approved the final version for submission. All authors contributed to the article and approved the submitted version.

## Conflict of Interest

The authors declare that the research was conducted in the absence of any commercial or financial relationships that could be construed as a potential conflict of interest.
